# Examining the Preliminary Effectiveness and Acceptability of a Web-Based Training Program for Australian Secondary School Teachers: Pilot Study of the BEAM (Building Educators’ Skills in Adolescent Mental Health) Program

**DOI:** 10.2196/29989

**Published:** 2021-10-22

**Authors:** Belinda L Parker, Melissa Anderson, Philip J Batterham, Aimee Gayed, Mirjana Subotic-Kerry, Melinda R Achilles, Cassandra Chakouch, Aliza Werner-Seidler, Alexis E Whitton, Bridianne O’Dea

**Affiliations:** 1 Black Dog Institute Randwick, NSW Australia; 2 Faculty of Medicine University of New South Wales Sydney Kensington, NSW Australia; 3 Centre for Mental Health Research Australian National University Canberra, ACT Australia

**Keywords:** mental health, training, high school teachers, youth, mental health programs, secondary schools

## Abstract

**Background:**

Secondary schools are increasingly supporting adolescents’ mental health and well-being, yet many teachers report that they lack the skills and confidence to do so. Building Educators’ skills in Adolescent Mental Health (BEAM) is a web-based training program developed to improve secondary school teachers’ knowledge and confidence in caring for students’ mental health.

**Objective:**

This pilot study examined the preliminary effectiveness and acceptability of the BEAM program for improving mental health knowledge, attitudes, confidence, helping behaviors, and psychological distress among secondary school teachers.

**Methods:**

A single-arm pilot trial was conducted from July to December 2019 among secondary school teachers located in New South Wales, Australia, who were employed in leadership positions responsible for managing student well-being (ie, Year Advisors). Participants had access to the BEAM program for 6 weeks. Self-report surveys, delivered at baseline, postintervention (6-weeks post baseline) and 3-month follow-up (19 weeks post baseline) were used to measure changes in training outcomes. Acceptability was assessed by program use, barriers, satisfaction, and participants’ perceptions of program effectiveness.

**Results:**

A total of 70 secondary school teachers took part (mean age 36.5 years, SD 9.41 years, range 24-60 years). Significant improvements in confidence were reported at postintervention and 3-month follow-up. Significant improvements in helping behaviors were reported at 3-month follow-up only. There was also a significant reduction in psychological distress at postintervention. Participants agreed that the program content was easy to understand and relevant, but program completion was challenged by lack of time, competing priorities, and forgetfulness.

**Conclusions:**

Findings indicated that a web-based training program may be beneficial for improving secondary school teachers’ abilities to care for students’ mental health; however, program modifications are required to increase training completions.

**Trial Registration:**

Australian New Zealand Clinical Trials Registry (ANZCTR) ACTRN12619000821190, Universal Trial Number U1111-1232-7680; https://www.anzctr.org.au/Trial/Registration/TrialReview.aspx?id=377529

## Introduction

### Background

Adolescence is a key period for the development of mental illness, with over half of all mental health problems first emerging before the age of 18 years [1]. Secondary schools are increasingly providing mental health care and support to adolescent students, primarily owing to the accessible nature of educational settings for service provision and the functional impacts of mental illness on social development and academic achievement. There are over 2800 secondary schools in Australia, enrolling over 2 million young people and employing more than 140,000 full-time teachers. One-third (n=830) of these schools are in New South Wales (NSW), where the average school size is 700 students with 58 teachers [2,3]. Population surveys in Australia and the United States [4] have confirmed the use of school services for mental health problems among youth, with service utilization rates similar to those of traditional health care settings [5]. Secondary schools can enable the early identification of mental illness, [6-9] as many Australian teachers are the first to identify emotional problems in students and initiate help-seeking [5]. However, many teachers report a lack of skills and confidence in recognizing and responding to students’ mental health needs [10,11] and few have received specialized training in this domain [8,12]. Many teachers have reported that additional management of student well-being has led to greater workload and stress [8,13,14]. In 2020, specialized mental health training was mandated for all NSW teachers [15], further confirming the need for these skills. Workplace learning and development opportunities may improve teachers’ abilities to care for students’ mental health and reduce the associated stress [16,17]. However, there are limited evidence-based training programs currently available.

Three recent systematic reviews on youth mental health training for secondary school teachers have indicated a lack of evidence-based programs [18-20]. Anderson et al [18] found that only 6 programs had been evaluated, and while all improved the mental health knowledge of the participating teachers, none increased actual helping behavior toward students (eg, referring or recommending a student seek professional help). Four of the reviewed training programs were delivered through didactic instruction (the remaining 2 were a combination of face-to-face and web-based components) and required teachers to be absent from school duties, likely limiting the uptake [18]. Furthermore, only 1 program was evaluated within the Australian context [21,22]. Similarly, Yamaguchi et al [19] noted that while most programs observed an improvement in knowledge, attitudes, confidence, and behavior, the quality of studies was low [19]. These findings were further confirmed by Ohrt et al [20]. Taken together, these reviews suggest that a focus on mental health literacy alone is unlikely to elicit behavior change. There is a clear need for high quality programs that are evidence-based, flexible in delivery, and effective for improving teachers’ confidence and skills in the domain of student mental health. Web-based mental health training has been shown to be effective in workplace settings [23], and may provide a delivery model that is scalable and accessible for school settings.

**Figure 1:**
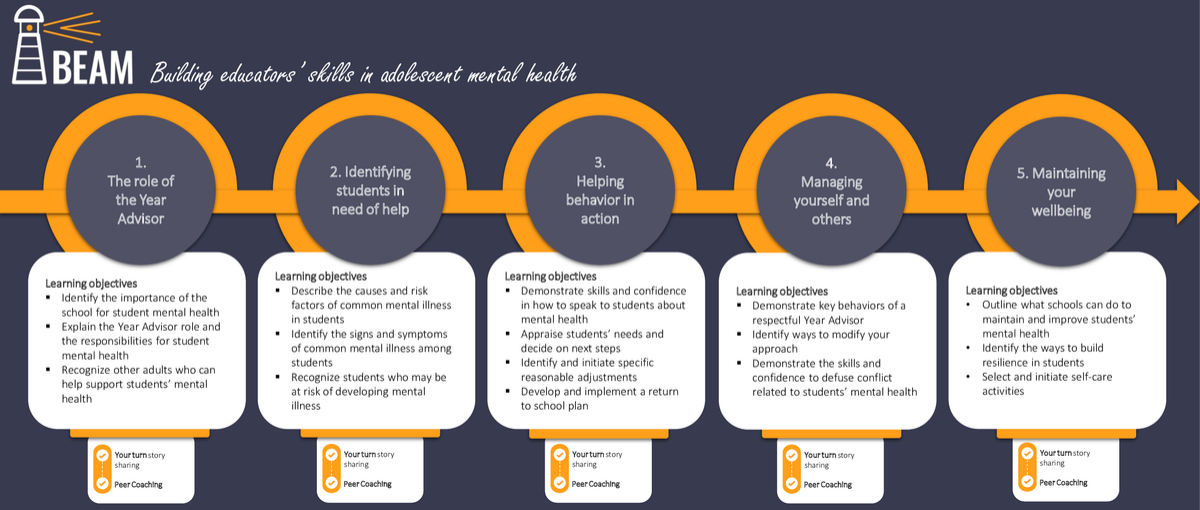
Overview of the Building Educators’ skills in Adolescent Mental Health (BEAM) training program.

The Black Dog Institute has developed a web-based training program that aims to improve secondary school teachers’ knowledge, confidence, and skills in recognizing and supporting students with mental health problems. Delivered over 6 weeks, the Building Educators’ skills in Adolescent Mental Health (BEAM) program combines self-directed content with in-person peer coaching activities and printable resources. The program consists of 5 topics that include educational information, quizzes, blog-style story-sharing, and case studies (Figure 1). Each topic is complemented by a peer coaching component that aims to help teachers contextualize the program, build professional relationships, self-reflect, and learn from those with different expertise and experience [24,25]. Topics are completed in sequential order and are not unlocked until the peer coaching activities are completed. However, topics can be completed at any pace during the 6 weeks with no limit on topics completed per week.

BEAM was developed in partnership with a lived experience advisory group of 12 teachers [12], and the delivery format was adapted from the evidence-based manager-training program HeadCoach [23,26]. HeadCoach is designed to help managers to better understand and support the mental health needs of their staff [23]. An evaluation of HeadCoach demonstrated positive effects on managers’ confidence and workplace practices for mental health, with significant improvements in responsive and preventative behaviors toward staff [23]. BEAM includes similar persuasive techniques (eg, reminders, feedback, theory, and practical advice) to target engagement [27] and tunneling (eg, program modules are presented in a structured and sequential manner) to guide program completion [28,29]. As such, the BEAM program can be completed at a time and place that is convenient for teachers. However, this web-based training model for student mental health is the first of its kind to be delivered in Australian secondary schools. It is unclear whether teachers are open to this type of training, and whether a self-directed program is effective for improving outcomes and behavior related to student mental health.

### Objectives and Hypotheses

This study examined the preliminary effectiveness and acceptability of the BEAM program for improving mental health knowledge, attitudes, confidence, and helping behaviors among secondary school teachers in NSW, Australia. It was hypothesized that participating teachers would report improvements in knowledge, attitudes, confidence, and helping behaviors at postintervention. This study also examined teachers’ psychological distress to determine whether the training program was associated with positive improvements in the mental health of teachers themselves. Program acceptability was examined by measures of program use, barriers to use, and training satisfaction. Our results provide important information about the feasibility of using a training platform that combines web-based and offline learning to address shortages in teachers’ professional development in student mental health.

## Methods

### Design

An uncontrolled, single-arm pilot study was conducted with outcome measures assessed at baseline, postintervention (6 weeks after baseline), and 3-month follow-up (19 weeks after baseline). The study was approved by the University of New South Wales (UNSW) Human Research Ethics Committee (HC190047), the NSW State Education Research Advisory Process (SERAP 2019048), and the Sydney, Parramatta, and Maitland-Newcastle Catholic Dioceses. This trial was registered with the Australian New Zealand Clinical Trials Registry (ACTRN12619000821190) and assigned a Universal Trial Number (U1111-1232-7680).

### Participants

The current study targeted secondary school teachers from NSW, who were employed in a teaching role with increased responsibility for the mental health of students aged between 12 and 18 years (eg, Year Advisor or equivalent, Student Coordinator, Head of Year). These teachers were identified as requiring significant skills in student mental health as they acted as informal case managers for students by offering support when needed and liaising with others about school performance. Secondary school teachers were eligible to participate in the current study if they were (1) employed full-time for the duration of the study in a teaching role that had responsibility for student well-being, (2) able to obtain support and signed consent from their school principal, and (3) able to participate alongside at least 1 other equivalent colleague from their school to enable completion of the peer coaching.

### Recruitment and Consent

Recruitment was undertaken between June 17 and July 19, 2019, in NSW. The study was advertised in 9 School-Link newsletters (ie, a state government service that connects schools with local mental health services). The study was also advertised on the Black Dog Institute’s website and Facebook page. Teachers were directed to the study website where they completed the eligibility screening and registered with their name, school, and email address, and downloaded the participant information statement and consent forms (PISCFs). Eligible teachers were advised to consult with their school principal and colleagues and email the completed PISCFs to the research team.

### Procedure

Once eligibility was confirmed and consent forms were returned, participants were invited to complete the baseline survey. Participants who did not attempt or complete the baseline survey within 7 days were withdrawn from the study. Upon completion of baseline, participants were given access to the BEAM program for 6 weeks. The BEAM program was accessible on internet-enabled computers only. Modules were completed sequentially, and all activities were mandatory. The program provided automatic feedback on incorrect responses and this content was not reviewed by the researchers. Responses to the peer coaching activities were submitted to the research team through the program and researchers provided feedback via email within 3 days of submission. Participants were sent 4 reminders to use the program. At 6 weeks post baseline, participants were invited to complete the postintervention survey. At 3-month follow up (ie, 19 weeks post baseline), participants were invited to complete the follow-up survey, and those who completed it were reimbursed with an Aus $50 (US $36.26) e-gift voucher, irrespective of whether they completed the BEAM program.

### Measures

#### Demographics and Background Characteristics

At baseline, participants reported their gender and age, teaching experience, duration in current role, and employment at current school (all reported in years). Participants also reported the location of their current school (metropolitan, regional, or rural/remote), the school funder (government or nongovernment), and gender type (single-sex or coeducational). Participants reported their current level of training in student mental health (none, limited, moderate, or extensive). Participants were asked to rate the importance of receiving mental health training and their confidence in web-based programs for satisfying their training needs. Items were answered using a 5-point Likert scale ranging from not at all (1) to extremely (5).

#### Mental Health Knowledge

A 6-item adapted subscale of the Mental Health Knowledge Schedule (MAKS) [30] scale was used to measure mental health knowledge. The first 6-item subscale assessed literacy (eg, “most students with mental health problems want to complete their schooling”) and were answered on a 5-point Likert scale ranging from strongly disagree (1) to strongly agree (5). Items were summed to yield a total score ranging from 6 to 30. Higher scores indicated greater mental health literacy. As the MAKS consists of multidimensional items that examine different mental health-related domains, calculating and reporting the subscale Cronbach α has been considered inappropriate [30].

#### Mental Health Attitudes

A modified version of the Depression Stigma Scale–Personal Stigma subscale [31] was used to measure teachers’ attitudes toward students with mental health problems. Participants were asked to rate how much they agreed with 9 statements (eg, “students with a mental illness could snap out of it if they wanted”) using a 5-point Likert scale ranging from strongly disagree (1) to strongly agree (5). Items were summed to yield a total score, ranging from 9 to 45. Higher scores indicated greater levels of stigma. In this study, Cronbach α=.82.

#### Confidence in Helping Behaviors

An adapted version of the Confidence in Helping subscale [32] was used to measure participants’ self-confidence in their ability to recognize, refer, and support students with mental health problems. Participants were asked to rate their confidence in dealing with 15 mental health scenarios (eg, “recognizing a student with mental health problems”). Items were answered using a 5-point Likert scale ranging from not at all confident (1) to very confident (5). Items were summed to yield a total score ranging from 15 to 75. Higher scores indicated greater levels of confidence. In this study, Cronbach α=.95.

#### Frequency of Helping Behaviors

A 14-item adapted version of the Help Provided to Students questionnaire [21] was used to measure the frequency of helping behaviors for mental health among teachers. Participants were asked to indicate how often, in the past 2 weeks, they had engaged in 14 helping behaviors (eg, “reached out to a student with mental health problems”). Items were answered using a 4-point scale (never, once, occasionally, or frequently). Items were summed to yield a total score ranging from 14 to 56. Higher scores indicated more frequent helping behaviors. In this study, Cronbach α=.89.

#### Psychological Distress

The self-report 5-item Distress Questionnaire-5 (DQ5) [33] was used to measure teachers’ psychological distress. Items were answered using a 5-point Likert scale ranging from never (1) to always (5). Items were summed to yield a total score ranging from 5 to 25. Higher scores indicated greater psychological distress with a score of 14 indicating the possibility of a mental health condition [33]. Completion of the DQ5 was optional, since it was not a primary outcome of the training program. The DQ5 has high internal consistency and convergent validity [33,34]. In this study, Cronbach α=.84.

#### Perceived Effectiveness

At postintervention, participants were asked to rate (using 4 separate items) the extent to which they believed the BEAM program increased their confidence, skills, and approach to students’ mental health needs, and whether the program met their training needs. Items were answered using a 5-point Likert scale ranging from not at all (1) to entirely (5).

#### Program Use and Barriers

Program use was measured by the number of topics completed. As topics were sequential, participants could not progress through the program without completing the peer coaching. Therefore, topics were deemed completed when a participant submitted the corresponding peer coaching activity. Barriers to program use were assessed at postintervention. Participants were asked to report whether they had experienced any items from a list of 11 barriers to program use (eg, “forgot about the program” and “the program wasn’t a priority,” answered with “yes” or “no”).

#### Program Acceptability and Satisfaction

Acceptability was measured at postintervention using a 13-item questionnaire that was adapted for the BEAM program [35,36]. Participants were asked to rate the extent to which they agreed with 13 statements (eg, “the content was easy to understand” and “the program activities were engaging”). Items were rated on a 5-point Likert scale ranging from strongly disagree (1) to strongly agree (5). Satisfaction was measured at postintervention using 3 items. Participants were asked to rate how satisfied they were with the program, the likelihood of future use, and the likelihood of recommending the program to others. Items were rated on a 5-point Likert scale ranging from not at all (1) to entirely (5).

### Data Collection and Analysis

Program and study data were collected and stored securely using the Black Dog Institute Research Engine hosted on the University of New South Wales servers in Australia. Data was then downloaded into Microsoft Excel and exported into SPSS (version 27.0, SPSS Inc) for analysis. The final sample for analysis consisted of the participants who completed the baseline assessment. At postintervention and 3-month follow-up, participants’ responses were included in the analyses regardless of whether they completed all questionnaires. Basic descriptive statistics, including means and standard deviations were calculated and reported for participant and school characteristics, and acceptability items. To estimate the pre- and postintervention effects of the program on participants’ mental health knowledge, confidence, attitudes, helping behaviors, and psychological distress, mixed models repeated measures analysis of variance were conducted, with time as a main effect. Statistical significance was set at *P*<.05 (2-tailed). We conducted *t* tests to compare mean differences in acceptability, satisfaction, and perceived effectiveness scores between program completers (ie, participants who completed the entire program) and noncompleters.

## Results

### Participants

**Figure 2:**
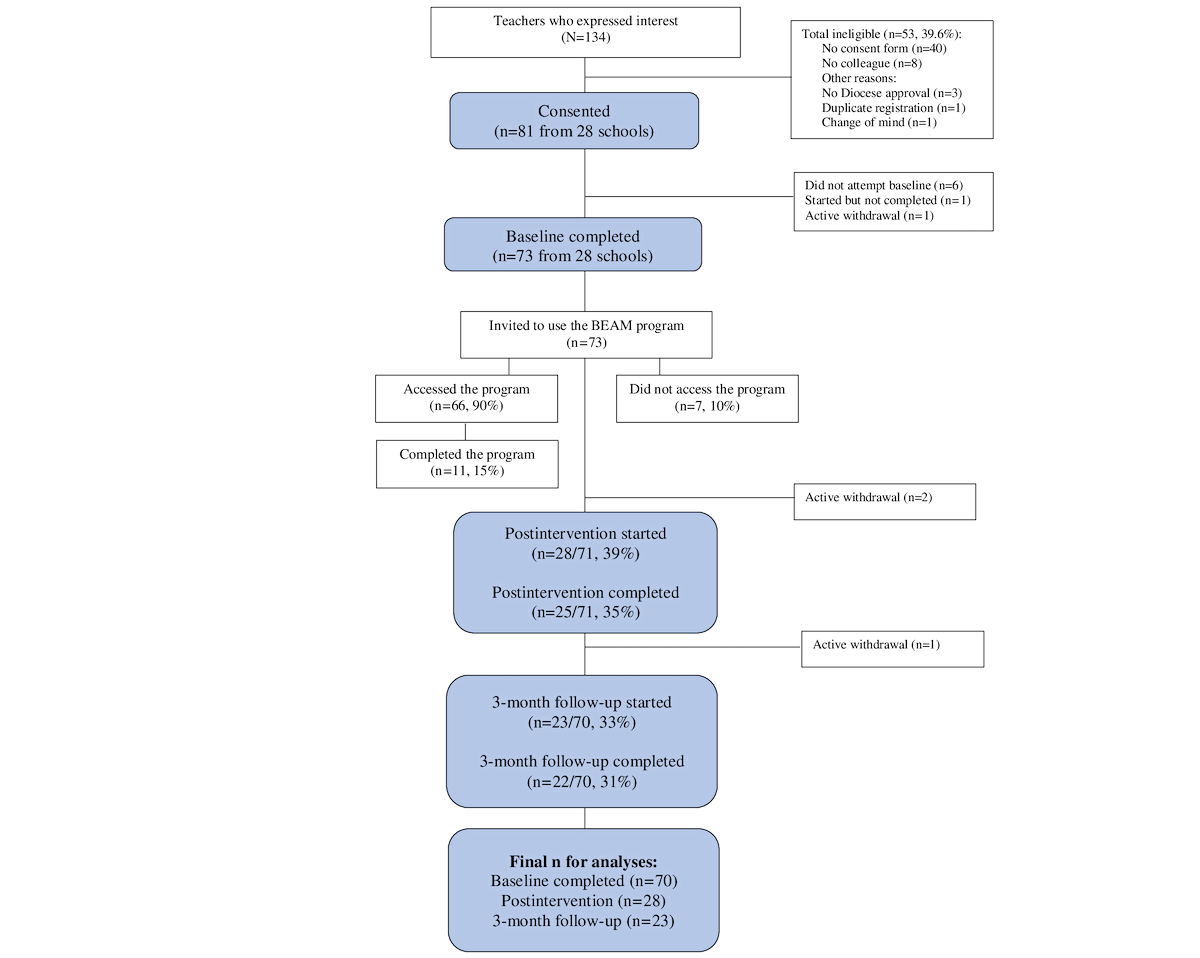
CONSORT (Consolidated Standards of Reporting Trials) diagram of study recruitment and flow. BEAM: Building Educators’ skills in Adolescent Mental Health.

A total of 134 teachers expressed interest in the study. Of these, 81 (60.4%) teachers from 28 schools obtained consent from their principal and were invited to complete the baseline survey (Figure 2). Table 1 presents the participant characteristics for the final sample at baseline (N=70).

At baseline, 40% (28/70) of the sample reported having had “nil” or “limited” prior training in student mental health and 60% (42/70) reported having had “moderate” or “extensive” prior training. All participants believed that mental health training was “moderately” (5/70, 7%) or “extremely” (65/70, 93%) important. The majority (51/70, 73%) felt “moderately” or “extremely confident” that a web-based program could meet their mental health training needs: 19% (13/70) were “neutral” and 9% (6/70) were “somewhat confident.”

**Table 1:** Sample characteristics at baseline (N=70).

Characteristics		Values
Females, n (%)		49 (70)
School funder: government, n (%)		68 (97)
School type: coeducational, n (%)		63 (90)
**School location, n (%)**		
	Metropolitan	21 (30)
	Regional	29 (41)
	Rural/remote	20 (29)
Age (years), mean (SD); range		36.47 (9.41); 24-60
Experience in current role (ie, Year Advisor) (years), mean (SD); range		3.33 (2.97); 0-15
Experience as a secondary school teacher (years), mean (SD); range		9.62 (6.82); 2-36
Duration of employment at current school (years), mean (SD); range		6.23 (4.40); 1-23

### Preliminary Effectiveness

There were no significant changes in mental health knowledge or attitudes at postintervention or 3-month follow-up (Tables 2 and 3). Significant increases in confidence were found at postintervention (Cohen *d*=0.60) and 3-month follow-up (Cohen *d*=0.53). There was also a significant increase in the frequency of helping behaviors at 3-month follow-up and a significant reduction in participants’ psychological distress at postintervention (Cohen *d*=0.33). Multimedia Appendix 1 provides further information regarding each measure.

When asked about their perceptions of training effectiveness at postintervention, the mean scores indicated that on average, participants rated the training program as “somewhat” (ie, score of 3) effective for improving their confidence, skills, and approach to student mental health (Table 4).

**Table 2:** Outcome measures at each time point.

Measures^a^	Baseline score, mean (SD)	Postintervention score, mean (SD)	3-month follow-up score, mean (SD)
Mental health knowledge	23.14 (2.27)	23.50 (2.24)	24.48 (2.71)
Mental health attitudes	13.67 (4.01)	13.71 (3.75)	14.86 (7.43)
Confidence in helping behavior	52.04 (10.68)	58.44 (9.23)	63.32 (6.37)
Frequency of helping behavior	35.37 (7.37)	36.46 (7.77)	41.68 (7.42)
Psychological distress	11.25 (3.78)	10.00 (3.39)	10.71 (3.23)

^a^Owing to attrition, the number of participants at postintervention varied: mental health knowledge, n=28; mental health attitudes n=28; confidence in helping behavior, n=27; frequency of helping behaviors, n=26; and psychological distress, n=21. Similarly, at follow-up: mental health knowledge, n=23; mental health attitudes, n=22; confidence in helping behavior, n=22; frequency of helping behaviors, n=22; and psychological distress, n=21.

**Table 3:** Estimates of change in outcomes at postintervention and 3-month follow-up based on mixed model repeated measures analysis of variance.

Outcomes	Postintervention vs baseline				3-month follow-up vs baseline			
	Estimate (SE)	*t* test (*df*)	*P* value	Cohen *d*^a^	Estimate (SE)	*t* test (*df*)	*P* value	Cohen *d*^a^
Mental health knowledge	0.41 (0.29)	1.45 (*38.03*)	.16	0.2	0.59 (0.38)	1.58 (*31.04*)	.13	0.4
Mental health attitudes	0.14 (0.61)	0.23 (*23.35*)	.82	0.01	1.25 (1.37)	0.91 (*23.99*)	.37	0.3
Confidence in helping behavior	6.83 (1.54)	4.44 (*30.24*)	<.001^b^	0.6	9.79 (1.29)	7.54 (*33.22*)	<.001^b^	0.5
Frequency of helping behavior	1.09 (1.00)	1.09 (*28.62*)	.28	0.2	4.98 (1.34)	3.73 (*23.90*)	.001^b^	0.7
Psychological distress	–1.09 (0.41)	–2.66 (*21.66*)	.01^b^	0.3	0.08 (0.45)	0.17 (*19.97*)	.87	0.1

^a^Cohen *d* effect sizes were calculated as the difference between observed means at postintervention (or follow-up) and baseline, divided by the SD at baseline.

^b^Significant at *P*<.05.

**Table 4:** Participants’ perceived effectiveness of the training program for increasing confidence, skills, approach, and meeting training needs at postintervention (n=25).

Measures	Values, mean (SD)
The extent to which the program increased your confidence to support students’ mental health	3.24 (0.88)
The extent to which the program increased your skills to support students’ mental health	3.32 (0.90)
The extent to which the program improved your approach to supporting students’ mental health	3.28 (0.90)
The extent to which the program met your mental health training needs	3.28 (0.84)

### Program Use and Barriers

**Figure 3:**
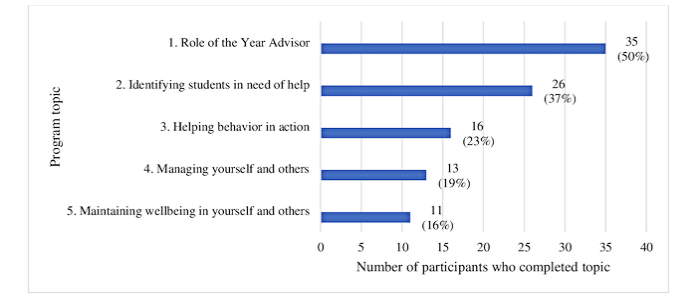
Program use: Sequential topic completion among participants (N=70). Note: Topics were sequential and locked. New topics could not be started unless the peer coaching activity was submitted to the program.

A total of 94% (66/70) of the baseline sample accessed the first topic and 50% (35/70) completed it. There was a steady decrease in topic completions over time: 16 (23.9%) participants completed half the program (ie, ≥3 topics) and 11 (16%) completed the entire program (Figure 3). Lack of time, competing priorities, and forgetfulness were reported as the common barriers to program completion. Lack of engagement with content, disruptions to working relationships, and taking leave were the least common barriers to program use (Table 5).

**Table 5:** Barriers to program use (n=26).

Barriers	Yes, n (%)
I didn’t have enough time to complete the program	22 (85)
The program wasn’t a priority	15 (58)
I forgot about the program	14 (54)
I had problems with my Internet connection or device access	4 (15)
I didn’t have another colleague to complete peer coaching activities	3 (12)
I had problems with accessing the program	2 (8)
I had concerns about the privacy and security of my data	2 (8)
Other reasons not listed	2 (8)
I went on leave from school/role	1 (4)
Program was disruptive to my working relationships	1 (4)
Program wasn’t engaging	1 (4)

### Program Acceptability and Satisfaction

Most participants rated the program content as easy to understand, relevant, practical, realistic, and helpful (Table 6). Participants gave lower ratings to the peer coaching and feedback components. Overall, participants were “somewhat” to “moderately” satisfied with the training program. *t* tests indicated no significant differences in the mean scores of acceptability, satisfaction, and perceived effectiveness between participants who completed the entire program (n=9) and those who did not (n=17) (*P*=.08-.98).

**Table 6:** Measures of program acceptability and satisfaction (n=26).

Measures			Score, mean (SD)
**Program acceptability**			
	The content was easy to understand	4.35 (0.56)	
	The content was relevant to my current role	4.27 (0.45)	
	The program content matched the learning objectives	4.23 (0.51)	
	The learning objectives were clear and realistic	4.19 (0.40)	
	The program structure was logical	4.15 (0.46)	
	The examples and suggestions were practical and realistic	4.12 (0.52)	
	The content was helpful for my current role	4.08 (0.56)	
	I had opportunities in the program to practice the skills learned	4.04 (0.72)	
	The program activities were engaging	3.65 (0.69)	
	The feedback I received throughout the program was constructive	3.62 (0.80)	
	The peer coaching activities were helpful	3.58 (0.76)	
	I enjoyed the opportunity to complete the Share my Story activity	3.08 (0.94)	
	The content was confronting or distressing	2.12 (1.07)	
**Satisfaction (n=25)**			
	Overall, how satisfied were you with the program?	3.60 (1.00)	
	How likely are you to use the program again in the future?	3.56 (1.00)	
	How likely are you to recommend the program to others?	3.84 (1.03)	

## Discussion

### Principal Findings

This study examined the preliminary effectiveness and acceptability of a web-based training program for improving mental health knowledge, attitudes, confidence, and helping behaviors among secondary school teachers in NSW. Our findings indicate that the BEAM program may be beneficial for improving teachers’ confidence in, and the frequency of, helping behaviors toward their adolescent students with mental health problems, when measured with self-report scales. The BEAM program did not appear to be associated with improvements in mental health knowledge or attitudes, although this may be owing to the high baseline levels of experience and training within this cohort. The BEAM program may also be associated with a reduction in teachers’ psychological distress, although this would need to be replicated within a randomized controlled trial. Given that past evaluations of teacher training programs for mental health have not observed improvements in helping behavior or teachers’ well-being [18,19], these initial findings are promising. However, study attrition was high and program completion rates were low. Modifications to the training program and associated study design are needed for future trials.

### Program Acceptability

Program acceptability was high among the teachers who remained in the study at postintervention. These teachers viewed the training content as relevant, helpful, and engaging but were less favorable toward the peer coaching activities. Only half of all participants continued beyond the first topic, suggesting that the compulsory completion of the peer coaching activity may have halted progress for many participants. While there was no evidence to suggest that program completion was associated with greater acceptability and satisfaction, it is difficult to examine overall levels owing to the high study attrition rate. Future trials may benefit from removing the mandatory completion of the peer coaching activities to encourage more training completions. This would allow participants to complete the program at their own pace and lead to greater exposure to training content. While it is not yet clear which persuasive techniques are associated with better outcomes for web-based programs [37], engagement with the BEAM program may also be enhanced by greater tailoring of content (eg, message personalization and topic recommendations based on experience and interest), thus improving the quality and relevance of the feedback, increasing interactivity, and removing the sequential format. These changes may improve completion rates, perceived effectiveness, and overall acceptability [38].

A key aim of this project was to create a mental health training program for secondary school teachers, which was highly accessible, easily disseminated, and scalable but also engaging and effective. The use of a web-based platform enabled these goals; however, this study shows that 1 in 4 teachers were not confident that this type of program could meet their training needs. Some teachers may have felt that the skills needed to care for students’ mental health could not be taught (or learnt) via a self-directed web-based program. Given that other web-based workplace mental health training programs have been shown to be effective [23], some teachers may require additional messaging to address these attitudes prior to commencing the training. Embedding acceptance facilitation techniques (eg, video explainers, provision of research findings, and feedback on the changes in participants’ outcomes) may help increase confidence and uptake of this training format [39,40]. Additional feedback from peers, students, and parents about improvements in participants’ helping behavior may also help strengthen perceptions of training effectiveness. The results may also indicate some discordance between participants’ improvements on the self-report outcome scales and their ratings of perceived training effectiveness. This may suggest that teachers did not perceive the training to be immediately beneficial and instead require more time to transition their new skills into practice and reflect on these changes. This would suggest that future study designs should carefully examine the outcomes measures used and ensure that suitable follow-up periods are used.

### Limitations

The study was impacted by high attrition, for both survey completion and program use, thus limiting firm conclusions about program effectiveness and acceptability. It remains unclear whether participants’ attitudes towards the program or other external factors influenced dropout. A larger sample size, with a control group, would allow for these analyses. Of the 11 participants who completed the program, 9 (82%) also completed the postintervention. This may suggest that improvements in program completions may also improve study retention. Future studies would likely benefit from using incentives for all study assessments, thus increasing the study duration to give participants more time to access and complete the program, and modifying the program in accordance with the user feedback to sustain engagement [41]. The high number of teachers expressing interest demonstrates the need the for this type of training program; however, the requirement of signed principal consent and a coparticipating colleague likely hindered recruitment. Future trials may benefit from revising the consent process, eligibility criteria, and study dissemination process to enable greater participation. Different results may also be found when the program is evaluated among teachers who have not received prior mental health training or are not currently in self-selected roles delegated to caring for student well-being.

### Conclusions

This study indicates that the BEAM program may improve teachers’ confidence in caring for students’ mental health and increase the frequency of helping behaviors. The training program may also reduce teachers’ psychological distress. The program content was easy to understand and relevant, although barriers related to time, competing priorities, and forgetfulness inhibited program use. The results have indicated that some program modifications are necessary to increase completion. Taken together, the findings demonstrate the potential of a web-based training program for improving helping behavior and related outcomes among Australian secondary school teachers. A randomized controlled trial of the BEAM program will help determine genuine effects.

## Appendix

Multimedia Appendix 1 Mean (SD) values for all outcome measure items.

## Abbreviations

BEAM: Building Educators’ skills in Adolescent Mental Health
DQ5: 5-item Distress Questionnaire
MAKS: Mental Health Knowledge Schedule
NSW: New South Wales
PISCF: Participant Information Sheet and Consent Form
UNSW: University of New South Wales
