# Supplementary material for: Examining the Preliminary Effectiveness and Acceptability of a Web-Based Training Program for Australian Secondary School Teachers: Pilot Study of the BEAM (Building Educators’ Skills in Adolescent Mental Health) Program
Source: JMIR Ment Health. 2021 Oct 22;8(10):e29989. doi: 10.2196/29989 (PMC8571691; doi:10.2196/29989)
Supplement: Multimedia Appendix 1 [file mental_v8i10e29989_app1.docx]

Supplementary Table 1: Means and standard deviations for all outcome measure items.

| Measure | Item | Baseline  *M (SD)* | Post-test  *M (SD)* | 3-month follow-up  *M (SD)* |
| --- | --- | --- | --- | --- |
| Mental health knowledge |  | 23.14 (2.27) | 23.50 (2.24) | 24.48 (2.71) |
|  | 1.Most students with mental health problems want to complete their schooling | 3.70 (0.94) | 3.68 (0.94) | 4.04 (0.82) |
|  | 2. If a student had a mental health problem, I know what advice to give them to get professional help | 3.70 (0.73) | 3.89 (0.63) | 4.35 (0.65) |
|  | 3. Medication can be an effective treatment for students with mental health problems | 3.80 (0.79) | 4.11 (0.57) | 4.04 (0.82) |
|  | 4. Psychotherapy for example, talking therapy or counselling can be an effective treatment for students with mental health problems | 4.34 (0.59) | 4.43 (0.50) | 4.43 (0.59) |
|  | 5. Students with severe mental health problems can fully recover | 3.71 (0.85) | 3.86 (1.08) | 4.13 (0.97) |
|  | 6. Most students with mental health problems go to a healthcare professional to get help (reverse scored) | 3.89 (0.75) | 3.54 (0.92) | 3.48 (0.99) |
| Mental health attitudes |  | 13.67 (4.01) | 13.71 (3.75) | 14.86 (7.43) |
|  | 1. Students with a mental illness could snap out of it if they wanted | 1.50 (0.72) | 1.39 (0.50) | 1.64 (0.90) |
|  | 2. A mental illness is a sign of personal weakness | 1.19 (0.39) | 1.29 (0.53) | 1.55 (0.96) |
|  | 3. A mental illness is not a real medical illness | 1.20 (0.40) | 1.25 (0.44) | 1.41 (0.91) |
|  | 4. Students with a mental illness are dangerous | 2.23 (0.85) | 2.18 (0.77) | 1.73 (0.70) |
|  | 5. It is best to avoid students with a mental illness so that you don’t develop this problem | 1.16 (0.37) | 1.32 (0.82) | 1.41 (0.91) |
|  | 6. If I had a mental illness, I would not tell anyone | 2.10 (1.07) | 1.82 (0.82) | 2.09 (1.23) |
|  | 7. Seeing a mental health professional means you are not strong enough to manage your own emotions | 1.24 (0.58) | 1.25 (0.44) | 1.55 (0.91) |
|  | 8. If I had a mental illness, I would not seek help from a mental health professional | 1.59 (0.81) | 1.75 (1.08) | 1.82 (1.10) |
|  | 9. I don’t think treatment for a mental illness, provided by a mental health professional, would work | 1.47 (0.74) | 1.46 (0.64) | 1.68 (0.95) |
| Confidence in helping behavior |  | 52.04 (10.68) | 58.44 (9.23**)** | 63.32 (6.37) |
|  | How confident are you in… |  |  |  |
|  | 1. Recognising a student with a mental health problem? | 3.31 (0.89) | 3.70 (0.72) | 3.95 (0.65) |
|  | 2. Reaching out to a student with a mental health problem? | 3.59 (0.91) | 4.00 (0.68) | 4.32 (0.57) |
|  | 3. Having a conversation with a student about their mental health? | 3.71 (0.89) | 4.04 (0.71) | 4.36 (0.58) |
|  | 4. Listening to a student talk about their mental health? | 4.13 (0.80) | 4.44 (0.58) | 4.50 (0.67) |
|  | 5. Calming down mentally distressed students? | 3.57 (0.83) | 4.04 (0.65) | 4.14 (0.64) |
|  | 6. Referring or recommending a student with a mental health problem to seek help? | 4.03 (0.82) | 4.41 (0.57) | 4.59 (0.50) |
|  | 7. Supporting a student with a mental health problem? | 3.61 (0.86) | 4.15 (0.66) | 4.36 (0.58) |
|  | 8. Supporting a student who is suicidal or self-harming? | 2.93 (0.98) | 3.48 (0.89) | 3.82 (0.80) |
|  | 9. Talking to parents about a student’s mental health problem? | 3.27 (1.05) | 3.74 (0.94) | 4.14 (0.71) |
|  | 10. Talking to teachers and other school staff about a student’s mental health problem? | 4.04 (0.82) | 4.07 (0.87) | 4.55 (0.60) |
|  | 11. Dealing with difficult or challenging situations related to a student’s mental health problem? | 3.41 (0.89) | 3.74 (0.81) | 4.18 (0.59) |
|  | 12. Modifying school to help a student recover from a mental health problem? | 3.30 (1.07) | 3.78 (0.93) | 4.23 (0.81) |
|  | 13. Preparing a return to school plan for a student who has been absent due to a mental health problem? | 2.90 (1.25) | 3.44 (1.09) | 4.09 (0.97) |
|  | 14. Educating students and other staff about mental health problems? | 3.10 (1.08) | 3.63 (1.08) | 3.95 (0.95) |
|  | 15. Implementing preventative and resilience strategies for students? | 3.13 (0.95) | 3.78 (0.85) | 4.14 (0.56) |
| Frequency of helping behavior |  | 35.37 (7.37) | 36.46 (7.77) | 41.68 (7.42) |
|  | Thinking back over the past six weeks, how often have you… |  |  |  |
|  | 1. Recognised a student in need of help for their mental health? | 2.97 (0.80) | 3.00 (0.63) | 3.32 (0.57) |
|  | 2. Reached out to a student with a mental health problem? | 3.04 (0.77) | 3.04 (0.87) | 3.36 (0.79) |
|  | 3. Had a conversation with a student about their mental health? | 3.13 (0.78) | 3.12 (0.86) | 3.50 (0.60) |
|  | 4. Spent time listening to students’ problems? | 3.63 (0.54) | 3.54 (0.76) | 3.64 (0.49) |
|  | 5. Spent time calming students’ down? | 3.16 (0.79) | 3.12 (0.71) | 3.50 (0.60) |
|  | 6. Talked to a student about suicidal thoughts or self-harm? | 1.83 (0.87) | 2.04 (0.82) | 2.23 (1.07) |
|  | 7. Recommended or referred a student to seek professional help? | 2.73 (0.88) | 2.77 (0.76) | 3.32 (0.72) |
|  | 8. Consulted with a parent about a student’s mental health? | 2.21 (0.90) | 2.38 (0.98) | 2.95 (0.95) |
|  | 9. Consulted with other school staff about a students mental health? | 3.09 (0.68) | 3.04 (0.92) | 3.32 (0.57) |
|  | 10. Spoken to your cohort about mental health issues and wellbeing? | 2.27 (0.96) | 2.50 (1.10) | 2.77 (0.92) |
|  | 11. Modified school to help a student recover from a mental health problem? | 1.91 (0.93) | 2.08 (1.02) | 2.77 (0.97) |
|  | 12. Prepared a return to school plan for a student who has been absent due to a mental health problem? | 1.49 (0.70) | 1.62 (0.75) | 2.18 (1.10) |
|  | 13. Implemented preventative and resilience strategies for students? | 2.14 (0.82) | 2.31 (1.05) | 2.91 (0.97) |
|  | 14. Done anything else to respond to or support students’ mental health in your school? | 1.77 (0.97) | 1.92 (1.13) | 1.91 (1.27) |
| Psychological distress |  | 11.25 (3.78) | 10.00 (3.39) | 10.71 (3.23) |
|  | Thinking back over the past six weeks, how often have… |  |  |  |
|  | 1. Your worries overwhelmed you | 2.69 (0.96) | 2.24 (0.89) | 2.43 (0.81) |
|  | 2. You felt hopeless | 2.23 (0.96) | 1.71 (0.78) | 1.90 (0.77) |
|  | 3. You found social settings upsetting | 1.89 (0.91) | 1.71 (0.90) | 1.86 (0.85) |
|  | 4. You had trouble staying focused on tasks | 2.50 (0.98) | 2.43 (0.75) | 2.62 (1.07) |
|  | 5. Anxiety or fear interfered with your ability to do the things you needed to do at work or at home | 1.94 (1.05) | 1.90 (1.04) | 1.90 (0.62) |

Note. Due to attrition, *N* at post-test varied: Mental health knowledge (*n*=28); Mental health attitudes (*n*=28); Confidence in helping behavior (*n*=27); Frequency of helping behaviours (*n*=26); Psychological distress (*n*=21). Likewise, *N* at follow-up: Mental health knowledge (*n*=23); Mental health attitudes (*n*=22); Confidence in helping behavior (*n*=22); Frequency of helping behaviours (*n*=22); Psychological distress (*n*=21).
